# Gap selection and steering during obstacle avoidance in pigeons

**DOI:** 10.1242/jeb.244215

**Published:** 2023-01-23

**Authors:** Natalia Pérez-Campanero Antolín, Graham K. Taylor

**Affiliations:** Department of Biology, University of Oxford, 11A Mansfield Road, Oxford OX1 3SZ, UK

**Keywords:** Visually guided flight, Bird flight, Gap negotiation, Guidance law, Proportional navigation, Motion capture, *Columba livia*

## Abstract

The ability of birds to fly through cluttered environments has inspired biologists interested in understanding its underlying mechanisms, and engineers interested in applying its underpinning principles. To analyse this problem empirically, we break it down into two distinct, but related, questions: How do birds select which gaps to aim for? And how do they steer through them? We answered these questions using a combined experimental and modelling approach, in which we released pigeons (*Columbia livia domestica*) inside a large hall with an open exit separated from the release point by a curtain creating two vertical gaps – one of which was obstructed by an obstacle. We tracked the birds using a high-speed motion capture system, and found that their gap choice seemed to be biased by their intrinsic handedness, rather than determined by extrinsic cues such as the size of the gap or its alignment with the destination. We modelled the pigeons' steering behaviour algorithmically by simulating their flight trajectories under a set of six candidate guidance laws, including those used previously to model target-oriented flight behaviours in birds. We found that their flights were best modelled by delayed proportional navigation commanding turning in proportion to the angular rate of the line-of-sight from the pigeon to the midpoint of the gap. Our results are consistent with this being a two-phase behaviour, in which the pigeon heads forward from the release point before steering towards the midpoint of whichever gap it chooses to aim for under closed-loop guidance. Our findings have implications for the sensorimotor mechanisms that underlie clutter negotiation in birds, uniting this with other kinds of target-oriented behaviours including aerial pursuit.

## Teaser

**Summary:** The flight trajectories of pigeons steering through gaps between obstacles are well modelled by the same proportional navigation guidance law as describes the pursuit behaviours of birds and insects.

## INTRODUCTION

When B. F. Skinner proposed using pigeons to guide flying vehicles in World War II (Capshew, 1993), he may have been onto something. Pigeons have colonized complex, cluttered urban environments throughout the world, which they negotiate successfully at high speeds. They achieve this visually, aided by their panoramic (300 deg) field of view, and by visual processing some three times faster than a human's (Jones et al., 2007). However, rather than using operant conditioning to train pigeons to pilot vehicles by pecking at a screen as Skinner proposed, a better approach might have been to study the guidance algorithms by which they steer their flight. Here, we set out to do just that, using a combined experimental and modelling approach to investigate how pigeons steer towards gaps. This work is closely inspired by previous research on pigeons flying through a dense forest of vertical poles (Lin et al., 2014), but reduces the problem to its simplest level, by presenting the birds with a binary choice between an obstructed or unobstructed gap through which to fly.

Algorithmic approaches to the study of goal-directed behaviours (Hein et al., 2020) have been successful in explaining the detailed flight trajectories of pigeons negotiating clutter (Lin et al., 2014), and bats (Ghose et al., 2006), raptors (Brighton et al., 2017, 2021a,b; Brighton and Taylor, 2019) and flies (Fabian et al., 2018) intercepting prey. These studies use differential equations to build simple phenomenological models that are capable of accurately describing complex behavioural data. This approach models behaviour as a dynamical input–output relationship governed by a guidance law treating the animal's motion (e.g. its horizontal turn rate) as a control input that is commanded by feeding back the sensory output the behaviour produces (e.g. information on the target's relative position or motion). Different guidance laws will in general produce different behaviours. For instance, guidance laws using different sensory information typically generate trajectories from different families of curves, yielding variation that can be used for model selection and identification in relation to empirical data.

**Fig. 1.**
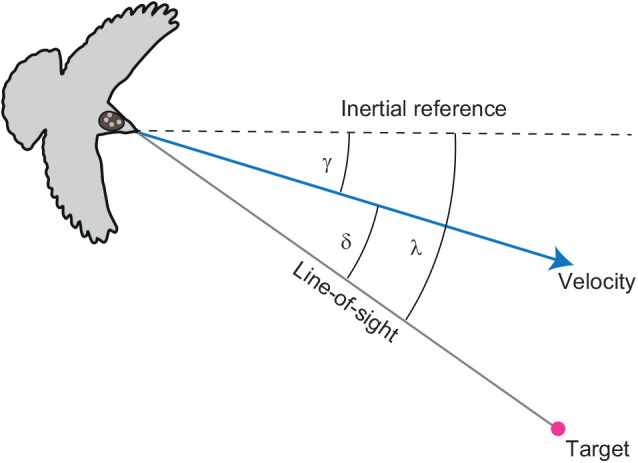
**Geometry of target-oriented guidance behaviour.** Definition sketch showing: the line-of-sight angle λ measured between the line-of-sight (grey line) from pigeon to target (magenta circle), and some arbitrary inertial reference direction (dashed line); the track angle γ measured between the bird's ground velocity vector (blue arrow) and the inertial reference direction (dashed line); and the deviation angle δ measured between the line-of-sight from bird to target (grey line) and the pigeon's velocity vector (blue arrow). The arrangement of the tracked headpack markers is shown schematically.

The sensory information that is most relevant to guiding target-oriented behaviour describes the direction of the line-of-sight vector connecting the subject to its target. This is characterised by two angles: the line-of-sight angle (λ), defined as the angle between the line-of-sight and some arbitrary inertial reference (e.g. true north); and the deviation angle (δ), defined as the angle between the line-of-sight and the subject's velocity vector (Fig. 1). Driving the deviation angle δ to zero causes the subject to fly directly at its target, whereas driving δ to some non-zero angle produces a spiralling approach. It can therefore make sense to use both the deviation angle δ and its time derivative 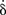 to control turning. In contrast, unless there is a reason to drive an approach towards some particular bearing, the line-of-sight angle λ is not itself used to control turning in target-oriented flight. Nevertheless, because λ remains constant for any pair of objects on a direct collision course, driving its time derivative 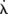 to zero leads naturally to interception. For this reason, the line-of-sight rate 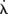 is often used to control turning in target-oriented flight.

Apart from shaping the resulting flight trajectory, the choice of what sensory information to use has implications for how it is obtained. For instance, if head movements are used to look directly at the target, then the output of the vestibular system can be used to estimate the line-of-sight rate 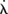. Therefore, identifying which input variables best model an animal's steering output has important implications for understanding the underlying physiological mechanisms. Moreover, because it is only the motion of the subject relative to its target that matters in target-oriented flight, the same guidance laws can be applied to both stationary and moving targets. This opens the possibility of unifying target chasing and clutter negotiation behaviours under one common algorithmic framework. To this end, we begin by reviewing the guidance laws that have been used to model target-oriented behaviours of both kinds to date.

### Algorithmic framework

Inspired by the types of controllers used in the engineering literature, early attempts to model the steering of animal flight (Collett and Land, 1978) looked for a proportional relationship between the subject's turn rate 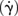 and the deviation angle (δ). The simplest form of this proportionality corresponds to the guidance law 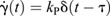, called pure or proportional pursuit (Brighton and Taylor, 2019), where *k*_P_<0 is the guidance gain, *t* is time and τ≥0 denotes a fixed delay. Pure pursuit drives the deviation angle δ towards zero, thereby causing the subject to aim its flight directly at its target. A simple variant called deviated pursuit drives the deviation angle δ towards some nonzero constant δ_0_ with 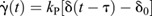. This causes flight to be aimed at a point ahead of a moving target, which promotes effective interception of a moving target (cf. pure pursuit, which leads directly to a tail chase), but produces a spiraling trajectory about a stationary target, which could help to steer flight around, rather than into, an obstacle (cf. pure pursuit, which leads directly to a collision).

Because the performance of a proportional controller can often be improved by adding derivative feedback, several studies have looked for the involvement of an additional derivative term 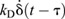, where 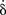 is the rate of change of the deviation angle and *k*_D_<0 is the associated guidance gain (Collett and Land, 1978; Lin et al., 2014). The addition of this derivative term anticipates the changes in deviation angle that are the basis of proportional pursuit, but commands high turning rates if the deviation angle changes rapidly, which can cause instability at high gain. Because derivative control has no inherent tendency to correct for any constant offset in the deviation angle, it is usually only used in combination with proportional control, but we also test it in isolation to aid in statistical inference. Adding an integral term to the controller is not expected to be useful in this context, given that driving the deviation angle δ to any small angle is sufficient to bring the subject to its target (see above).

Other plausible steering controllers command turning in proportion to the angular rate of the subject's line-of-sight to its target 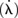, measured in an inertial frame of reference. The simplest form of this proportionality corresponds to the classical guidance law of homing missiles, 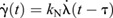, called proportional navigation (Brighton et al., 2017). This guidance law has been used to explain the attack trajectories of raptors (Brighton et al., 2017, 2021a,b; Brighton and Taylor, 2019; Kane et al., 2015), and effectively combines a proportional controller's tendency to correct a constant error signal with a derivative controller's tendency to anticipate changes in the error signal. It achieves this by driving a constant-bearing approach to the target, which typically leads to interception, rather than tail-chasing, of moving targets at *k*_N_>1. This guidance gain *k*_N_ is usually called the navigation constant and is conventionally denoted *N*, but we write it here as *k*_N_ for consistency with our notation for *k*_P_ and *k*_D_.

In contrast to proportional pursuit, proportional navigation is considered an optimal guidance strategy, in the sense that it can be tuned to minimise the steering effort needed to hit a non-manoeuvring target (Shneydor, 1998). Here, steering effort is defined as the sum of the squared lateral acceleration command, and non-manoeuvring means that the target is either stationary or flying in a straight line. These properties make proportional navigation an appealing candidate for gap-oriented steering, but previous research on gap aiming behaviours in birds has focused only on guidance laws involving the proportional-derivative guidance terms *k*_P_ and *k*_D_ (Lin et al., 2014). Proportional navigation can also be used to model deviated pursuit when *k*_N_=1, which matches the subject's turn rate 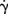 to its line-of-sight rate 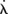, and therefore tends to keep the deviation angle δ constant at its initial value of δ_0_=δ(0) (see Fig. 1). We make use of this fact to avoid having to fit δ_0_ as a second free parameter in this single-input guidance law, and do not model deviated pursuit explicitly here (Table 1).

**Table 1.**
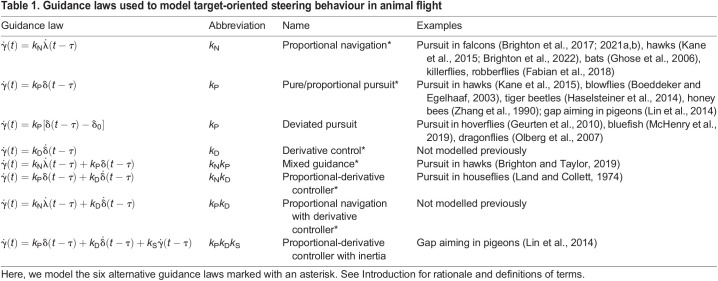
Guidance laws used to model target-oriented steering behaviour in animal flight

Different linear combinations of these *k*_P_, *k*_D_ and *k*_N_ terms have been used to formulate mixed guidance laws of the forms shown in Table 1. For example, mixed *k*_N_*k*_P_ guidance has been used to explain the attack trajectories of hawks pursuing manoeuvring targets (Brighton and Taylor, 2019), whereas proportional-derivative *k*_P_*k*_D_ guidance has been used to model pigeons steering through clutter (Lin et al., 2014). Finally, the *k*_P_*k*_D_ controller that Lin et al. (2014) used to model gap aiming behaviour in pigeons was originally tested with the addition of a delayed damping term 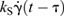 that we do not model here.

Whereas guidance laws can be used straightforwardly to model the target chasing behaviours of birds (Brighton et al., 2017, 2021a,b; Brighton and Taylor, 2019), using them to model clutter negotiation behaviour poses several new challenges. A key contribution of the pioneering work by Lin et al. (2014) was to treat clutter negotiation not as an obstacle avoidance behaviour, but as a gap targeting behaviour, thereby enabling its treatment as a classical guidance problem. Framing an animal's movement through a field of obstacles as a sequence of consecutive gap-aiming events (Lin et al., 2014) avoids the need to model more complex attractor–repeller type behaviours, but risks over-fitting if there are many obstacles, and hence multiple gaps, present. Moreover, a key challenge in applying this framework is the need to define what constitutes a target, and – following from this – the extent to which obstacle avoidance behaviour can be described as target-oriented movement at all. This is because an animal's perception of what constitutes a gap may be different to how an experimenter defines it (Baird and Dacke, 2016). How an animal perceives a gap will depend not only on the visual angles subtended by the objects in the environment (Lin et al., 2014), but also on background brightness (Baird and Dacke, 2016) and the subjective distance at which the animal treats an object as an obstacle relevant to gap choice. It follows that the gaps between obstacles are virtual constructs that are liable to change as an animal moves.

In this study, we therefore used a simplified experimental setup to identify the mechanisms of gap choice and gap steering independently. Using a forced binary choice protocol in which one of the gaps was partially obstructed allowed us to test whether the birds flew towards the gap providing the greatest clearance, or whether they used some alternative method of gap selection. Forcing the birds to manoeuvre towards one of two divergent positions also gave us sufficiently varied trajectory data to test alternative guidance laws modelling their steering (Table 1), and to test whether pigeons target the physical centre of the gap between obstacles as Lin et al. (2014) proposed.

## MATERIALS AND METHODS

### Subjects and training

We used *N*=12 homing pigeons (*Columba livia domestica* Gmelin 1789) aged from 2 to 10 years as subjects (Datasets 1 and 2), choosing this sample size to be at least twice that of related studies of gap negotiation behaviours in birds (Bhagavatula et al., 2014; Lin et al., 2014). The sex of these individuals is unknown, owing to the lack of unambiguous external sex characteristics in this species. The birds were reared at the John Krebs Field Station, Wytham, Oxford, UK, as members of a free-ranging population provided with *ad libitum* access to food and water in the home loft to which they returned voluntarily. The most experienced non-retired individuals of the population were selected for experiments in order to minimise distress to the animals.

Experiments took place over a 4 week period from mid-November to mid-December 2018. The birds were caught from their loft immediately before the experiment, and released by an experimenter in a large indoor flight hall with one of its ends open to the outside. The birds usually returned directly to their home loft, which was located within 80 m of the exit. Each subject was released in the empty flight hall on five consecutive days prior to experimentation, to ensure familiarity with the location and its relationship to the home loft before introducing any obstructions. All testing was approved by the Animal Welfare and Ethical Review Board of the University of Oxford's Department of Zoology (permit number APA/1/5/ZOO/NASPA), and we monitored the feather condition and flight behaviour of the birds throughout the study for any signs of stress.

### Experimental set-up and protocol

Experiments were undertaken in a flight hall measuring 20.2×6.1 m, with a minimum ceiling height of 3.8 m. The walls of the hall were covered with camouflage netting to provide homogeneous visual contrast. Flicker-free LED lights provided a mixture of direct 4000K illumination and indirect 5000K illumination at approximately 1000 lux, which was designed to mimic overcast morning or evening light conditions. Most of the back wall of the building comprised an open roller shutter door providing natural daylight illumination, presenting a much brighter scene than the interior of the flight hall. The ambient weather conditions and position of the sun's disc were a source of uncontrolled variation in brightness during the experiments. To minimise such variation, experiments were only undertaken during overcast days.

On each day of experiments, the pigeons were collected from their home loft, and fitted with a rigid plate attached to their head using eyelash glue (Duo Quick Set Strip Lash Adhesive Clear Tone). This was fitted just before the start of each recording session, and was removed at the end of the release. Each plate had a unique asymmetrical configuration of three or four 4 mm diameter spherical retro-reflective markers for motion capture purposes (Vicon Motion Systems Ltd, Oxford, UK). Each pigeon was taken out of its carrier box individually in the flight hall, held for 1 min at the release point to acclimatise, and then released to fly freely by the experimenter opening their hands. The next pigeon was only taken out of its carrier box once the preceding bird had exited the flight arena and was out of sight. After all the pigeons had been released, they were collected from the loft to be released again. On a typical test day, we would carry out three releases per pigeon. Testing was only conducted on clear days, and the markers were removed from the pigeons at the end of each day.

**Fig. 2.**
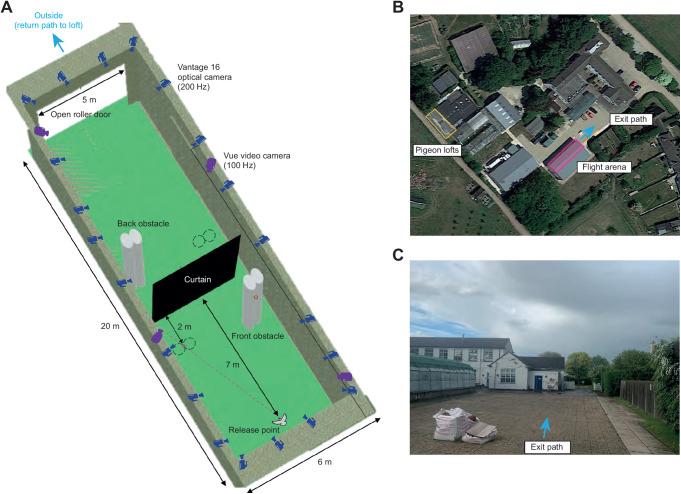
**Experimental setup.** (A) Flight arena viewed from above. Pigeons flew from the release point to the outside through an open roller door. The direct path to the roller door was obstructed by a curtain, creating a vertical gap to either side for the pigeon to fly through. A pair of cylindrical vertical obstacles was placed 2 m ahead of the curtain on either the left or the right side of the room, directly between the release point and the centre of the obstructed gap (front obstacle). The 7 m length of lab from the release point to the curtain defines the region of interest in which target-oriented steering flight was modelled. A second pair of obstacles placed behind the curtain (back obstacle) would not have been visible to the bird until after it had negotiated the gap, and is not considered here. Obstacle placement was randomised between the left and right sides at the start of each release; dashed circles represent their alternative placement. Note that the cylindrical obstacles appear distorted in this three-dimensional view. (B) Aerial view of experimental area relative to pigeon home lofts. (C) View out of experimental area from open shutter door. Note that buildings are visible to the left.

Pigeons were released at 1.2 m height approximately 2 m from the front wall of the hall, within ±1 m of the midline. From there, they flew through one of two floor-to-ceiling gaps created to either side of a heavy black curtain hung across the hall, approximately 7 m ahead of the release point (Fig. 2A). These symmetric vertical gaps were approximately 1.2 m wide, and hence up to roughly twice the wingspan of the birds. The aperture of the roller door was not visible to the birds at the point of release, but flying through either gap enabled the pigeons to see and reach this aperture, from where they could exit the building (Fig. 2C). Their home loft was located a short distance (approximately 80 m) behind and to the left of the building (Fig. 2B), so was occluded from view until the birds had exited the flight hall.

We placed a 4.0 m high obstacle in one of two fixed positions in each of the front and back sections of the hall (Fig. 2A). Each obstacle consisted of a pair of 0.3 m diameter white expanded polystyrene cylinders taped together side-by-side, giving high visual contrast against the background. The two obstacles were placed 2 m away from the curtain along the longitudinal axis of the flight arena to obstruct the most direct path from (i) the release point to either the right or left gap (front obstacle), and (ii) either the right or left gap of the curtain to the midpoint of the open shutter doors (back obstacle). We randomised the side on which the front and back obstacles were placed at the start of each release, and maintained the same obstacle positions for all the individuals on a given release to minimise the time that they were kept waiting in their carrier boxes. The back obstacle was not visible to the pigeon until it had reached the gaps on either side of the curtain, and is not considered further here. Here, we focus exclusively on the behaviour of the birds in the front section of the hall, between the release point and the curtain. Given that the curtain was located 9.0 m into the hall, we assume that both gaps would have appeared similarly bright to the birds were it not for the placement of the front obstacle (Fig. 2).

### Motion capture

We used an array of 22 high-speed motion capture cameras (Vantage 16, Vicon Motion Systems) and four reference video cameras (Vue, Vicon Motion Systems) to record the flights. Cameras were mounted 3.0 m above the floor on scaffolding fixed around the perimeter of the room. The cameras were arranged so that any marker within the recording volume would be visible to at least three cameras, which enabled automatic reconstruction of its 3D coordinates by the motion capture system. Sensor resolution for the Vantage cameras was 4096×4096 pixels at a 200 Hz frame rate and 1 ms shutter speed. A strobe unit on each camera emitted infrared light at 850 nm, which falls outside the visible spectrum of the birds (Remy and Emmerton, 1989); an optical filter blocked light at other wavelengths. For the Vue cameras, the sensor resolution was 1920×1080 pixels at a frame rate of 100 Hz and a shutter speed of 2 ms.

The camera system was calibrated at the start of each day of experiments. Experiments only went ahead after each camera had been calibrated to an image error accuracy of ≤0.4 pixels for the motion capture cameras, and ≤1.2 pixels for the reference video cameras. Flights were recorded using Vicon Nexus software, which was manually triggered for each pigeon release, with calibration error being checked at the start of each experimental session. The accuracy of the calibration decreased gradually through the day, but the mean three-dimensional reconstruction accuracy for all trials was 0.7 mm (Q1, Q3: 0.57, 1.26 mm). In addition to the retro-reflective markers fitted to the pigeons, we placed 6 mm diameter markers at fixed positions on the edges of the curtain, walls and obstacles, to record the positions of these features accurately for each trial. These markers were removed temporarily for calibration of the motion capture system.

The Vicon Nexus software outputs the 3D coordinates of all of the markers found within the imaging volume throughout the trial. We labelled each of the recorded markers using custom-written software in MATLAB v9.6 (MathWorks, Inc., Natick, MA, USA), which used clustering algorithms to group markers within and between frames (e.g. to distinguish markers on the pigeon from markers on the obstacles, based on their speeds). The marker labelling procedure was designed to eliminate the false positives that can arise from spurious reflections, but inevitably resulted in some data drop-out, particularly in the less-well-visualised part of the flight volume near the ceiling, and in the partially occluded regions of the flight volume close to the curtain. Marker positions were defined relative to the principal axes of the room, as determined through the calibration of the motion capture system. Any minor discrepancies in the alignment of this global axis system between trials were corrected by applying a Procrustes transformation to the camera coordinates prior to further analysis, so that the global axis definitions for all trials were the same.

### Flight trajectory analysis

We recorded a total of *n*=105 flights from the *N*=12 birds over 16 releases, having dropped any flights for which the recorded markers were insufficient for coordinate reconstruction, or where the bird did not fly to the exit point (e.g. perching on a camera instead). Of these, a total of *n*=95 flights from *N*=10 birds were judged suitable for detailed trajectory analysis in the sense that the pigeon was recorded flying through one of the gaps without landing or loitering. We determined pigeon head position as the mean position of the identified head markers, and fitted a quintic smoothing spline to remove the noise associated with occasional missing or merged markers. The smoothing tolerance was set such that the mean distance between the spline and the data was kept below the maximum span of the head markers, in order to preserve the detail of the head motion. Although the trajectory data are fully three-dimensional, we analyse only their horizontal components here, on the basis that the gaps we treat as the targets of the bird's guidance are extended objects in the vertical dimension.

### Steering controller simulations

We modelled the horizontal steering behaviour of our pigeons using the same trajectory simulation approach used to study target-oriented attack flights in hawks and falcons (Brighton et al., 2017, 2021a,b; Brighton and Taylor, 2019), but tested a broader set of candidate steering controllers comprising all single-input and two-input guidance laws involving linear combinations of *k*_N_, *k*_P_ and *k*_D_ (Table 1). We did not model deviated pursuit explicitly, because proportional navigation with *k*_N_=1 simulates a deviated pursuit trajectory in which the deviation angle δ remains constant at its initial value δ_0_=δ(0), thereby avoiding the need to treat the commanded deviation angle as another free parameter. Because the presence of an obstacle is expected to have modified the bird's perception of the obstructed gap, we only consider the subset of flights taken through the unobstructed gap for the purposes of modelling the steering controller. With this restriction, and to avoid over-fitting any very short recordings, we analysed only the subset of *n*=23 flights from *N*=10 individuals with ≥1 s of continuous recording during which the pigeons were tracked to within ≤1 m of the unobstructed gap through which they flew. It is important to note that this 1.0 s period of flight typically does necessarily extend to the point at which the bird finally reached the gap, owing to marker occlusion in the vicinity of the curtain.

For these *n*=23 flights, we fitted the candidate guidance laws under two alternative definitions of the target of the pigeons' gap-aiming behaviour: (i) the midpoint between the curtain edge and the wall (i.e. the point corresponding to the physical centre of the gap); and (ii) the point approximately half a wingspan (0.35 m) into the gap from the edge of the curtain (i.e. the point minimising the clearance with the wings fully spread). The former target definition maximises the bird's minimum clearance from the physical structures in its environment, whereas the latter minimises the clearance on the inside of the turn without risking a collision. Pigeons can fly through gaps as narrow as 0.11 m by closing their wings, but doing so compromises their efficiency and manoeuvrability (Williams and Biewener, 2015). Given that the physical size of the gap would not oblige the birds to close their wings, and given that we saw no evidence of them doing so, we opted for a target definition that would allow the birds to turn as tightly as possible without closing their wings.

Given the initial conditions recorded for each flight, we simulated the best-fitting flight trajectory commanded under the various guidance models shown in Table 1. For each steering controller, we estimated the guidance parameters and delay for a given flight, or collection of flights, by minimising the mean squared distance between the measured and simulated trajectories over all of the fitted sample points, which we report as the root mean square (RMS) error (ε). However, because the birds did not necessarily begin their target-oriented steering behaviour immediately upon release, we only use the data from the last 1.0 s of flight recorded before the pigeon entered the gap for the purposes of model selection and statistical inference; we later extend the simulated section of flight back in time to cover the entire recorded trajectory. Whereas we used the guidance law to simulate the bird's turning, we matched the simulated flight speed to the bird's measured flight speed, to ensure proper determination of the time derivatives of the line-of-sight angle λ and deviation angle δ.

To account for sensorimotor delay, we lagged the input variables by 0≤τ≤0.4 s. The upper end of this range of values exceeds the latency of the pectoralis muscles to the firing of the looming-sensitive neurons in the pigeon's tectofugal pathway by at least a factor of 4 (Wang and Frost, 1992), but is intended to accommodate the possibility that pigeon steering responses in flight might also be delayed by one wingbeat period (approximately 0.15 s inside the flight hall), as observed in turning flight (Ros and Biewener, 2017). Taking time *t*=0 as the first data point, we therefore modelled the bird's flight trajectory beginning from time *t*=τ_max_, to ensure that the same section of flight was simulated for all tested values of τ. We were thereby able to model each two-dimensional flight trajectory given knowledge of the pigeon's initial track angle and position, the time history of the pigeon's speed, and the location of the unobstructed gap formed by the edge of the curtain and the wall. See figshare for supporting code (https://doi.org/10.6084/m9.figshare.19285289).

### Statistical analysis

We use a generalized linear mixed effects (GLME) model with binomial link function in R (https://www.r-project.org/) to analyse the factors affecting left versus right gap choice over all *n*=105 flights and all *N*=12 birds, treating bird identity as a random effect and including the side of the obstructed gap as a fixed factor. We report the means of quantities estimated directly from the trajectory data together with their standard deviation (mean±s.d.) for the *n*=95 flights in which the pigeons flew through one or other gap without landing loitering, and use two-sample *t*-tests to test for differences in these means between samples. Note that we treat each flight as an independent data point when analysing the detailed properties of the trajectories, which means that some of the analyses risk pseudo-replication within birds.

For the subset of *n*=23 flights included in the guidance analysis, we use hat notation to refer to the least squares estimates of the guidance model parameters (e.g. 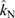 denotes a unique estimate of *k*_N_), and tilde notation to denote their median across all flights (e.g. 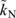 denotes the median value of 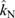 over all flights). We report these medians together with their associated 1st and 3rd quartiles (Q1, Q3), and use sign tests to test whether the fitted guidance constants were consistently positive or negative, or lower than some critical numerical value (see Discussion). We use Wilcoxon signed rank tests to test for differences in the median RMS error of the fitted guidance models, treating each flight as an independent data point. Unless stated otherwise, we report two-tailed *P*-values throughout.

## RESULTS

**Fig. 3.**
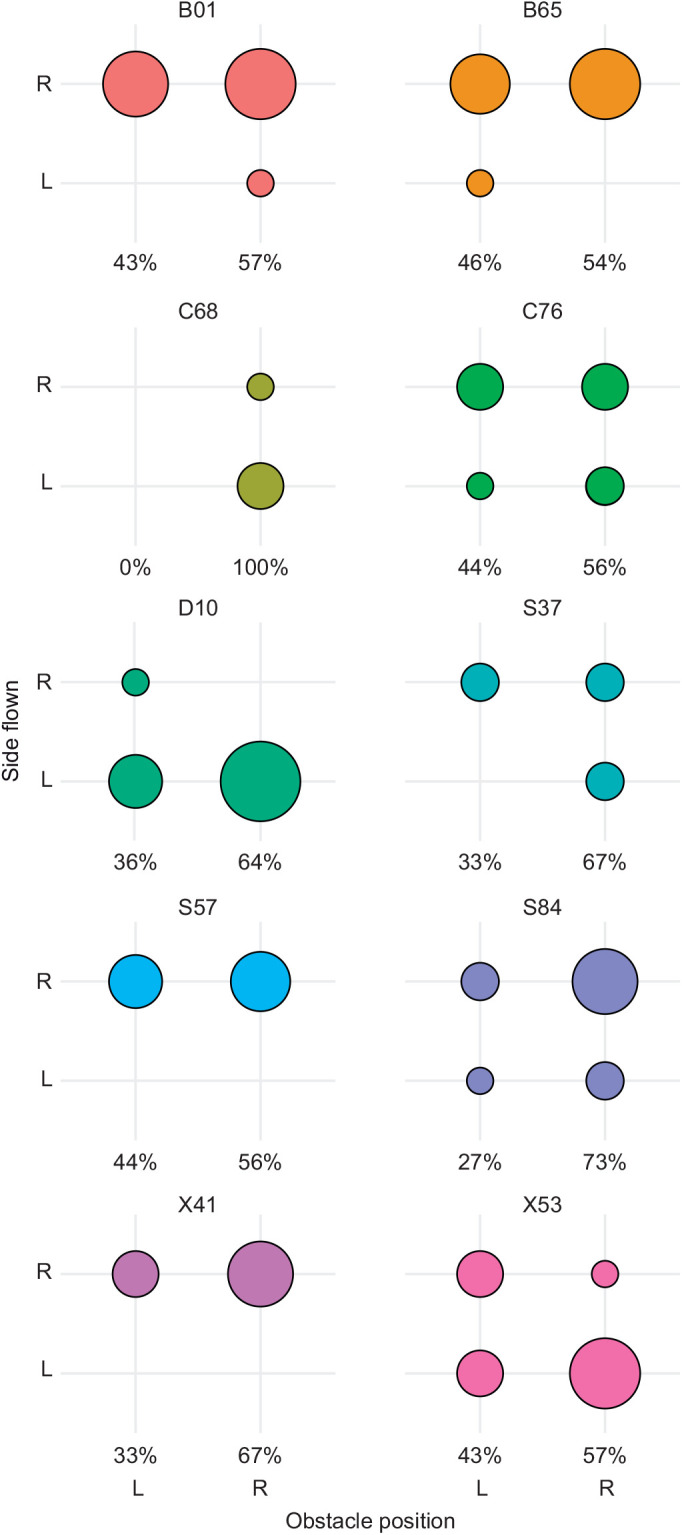
**Gap selection behaviour by individual.** Distribution of gap choice by different individuals, comparing the side flown with the side on which the obstacle was placed. Data are shown for all *n*=103 flights recorded from the *N*=10 birds included in the main guidance analysis; the single flights from the two birds that were not included in this main guidance analysis are not represented here (see Datasets 1 and 2 for details of all flights). Alphanumeric codes above each plot denote individual identity. The area of each plotted circle is shown proportional to the frequency of gap choice. Note that the individual pigeons were released sequentially in batches, and obstacle placement was randomised only once at the start of each release, to minimise the time during which individual birds were kept in their carrier boxes. The resulting percentages of left versus right obstacle placement are shown beneath each plot, corresponding to 10 releases with the obstacle on the right and six releases with the obstacle on the left; individual birds could not necessarily be included in all releases, owing to the need to catch them individually before each release. Most individuals display a clear preference for flying on one or other side of the curtain – most often the right-hand side. This idiosyncratic preference does not appear to be affected by obstacle placement (see Results).

**Fig. 4.**
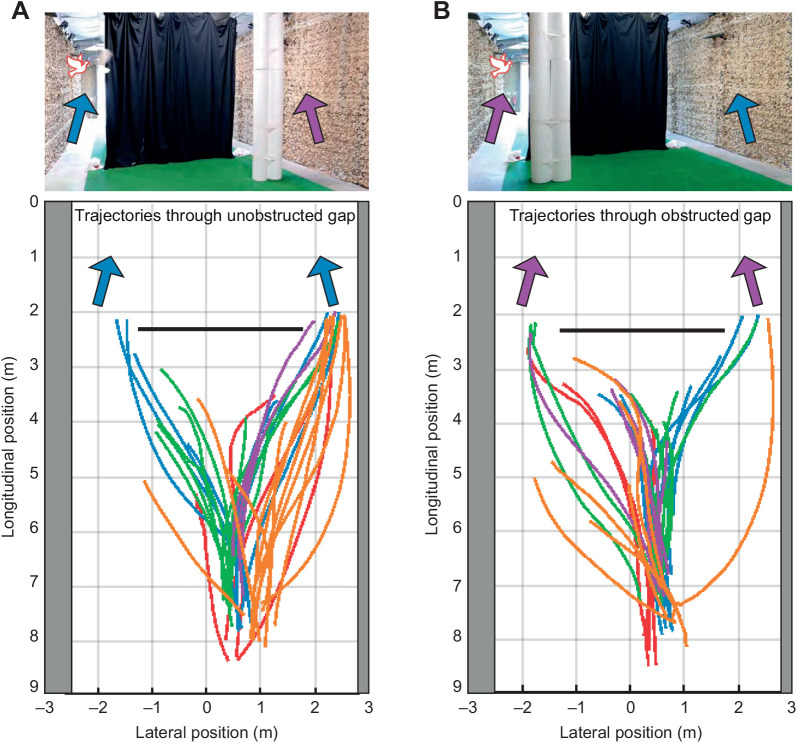
**Gap negotiation behaviour in pigeons.** Trajectory plots show the *n*=95 flight trajectories from the *N*=10 birds included in the main guidance analysis, selected as the subset of flights in which the birds flew through one or other gap without landing or loitering. Trajectories are colour-coded by individual and viewed from above. The upper panels illustrate pigeon flight behaviour schematically in relation to the experimental setup. Trajectories flown through the unobstructed gap (blue arrows) are shown in A; trajectories flown through the obstructed gap (purple arrows) are shown separately in B. Obstacle position was randomised at the start of each release, and pigeons were free to fly through either gap created by the curtain (solid black line). Note the right-handed bias that is apparent in the birds' choice of gap, and the fact that birds flying through the obstructed gap (B) appear to decide whether to diverge to the left or right later than birds flying through the unobstructed gap (A). Although the start points of the trajectories also appear biased to the right, this is because occlusion by the experimenter caused data dropout at the start of the flight, such that the handedness of the bird's choice may already be visible at the start of the recorded trajectory. Data dropout also occurs towards the ends of some trajectories, when the birds are flying close to the curtain or the ceiling.

Upon release, the pigeons flew in the direction of the open roller shutter door, even though its aperture was not directly visible from the release point, and despite this being in a different direction to the home loft. Most birds flew directly to the outside via one of the two gaps, but a few began circling in the section of the hall before the curtain. For the purposes of our analyses of gap selection (Fig. 3) and steering (Fig. 4), we only consider data collected between the release point and the curtain. The mean speed of flights through the unobstructed gap (4.0±0.8 m s^−1^) was significantly higher than for flights through the obstructed gap (3.6±0.7 m s^−1^; two-sample *t*-test: *t*_83_=2.37, *P*=0.02). These speeds are slow compared with the cruising speeds of pigeons flying in the open, which can exceed 10 m s^−1^ (Pennycuick, 1968), but are similar to the flight speeds observed previously under similarly cluttered experimental conditions (Lin et al., 2014).

### Pigeons select gaps on the basis of handedness, not size

The pigeons usually flew forwards upon release, before steering towards one of the two available gaps, resulting in a characteristic goblet-shaped distribution of flight trajectories (Fig. 4). We interpret this initial flight behaviour as an escape response, with the first unambiguous evidence of gap selection coming later in the flight. The pigeons flew through the unobstructed gap on 53% of the *n*=105 flights (overall odds of selecting the unobstructed gap: 1.14). The presence of an obstacle had no statistically significant effect on gap choice (GLME: *z*=1.84, *P*=0.07), so there is no evidence that the pigeons headed for the unobstructed, and hence larger, of the two gaps. However, comparing the detailed flight trajectories associated with flight through obstructed versus unobstructed gaps, it appears that birds flying through the obstructed gap may have made the decision to do so later than birds flying through the unobstructed gap, as the bifurcation of the goblet-shaped distribution occurs noticeably closer to the curtain for the subset of flights through the obstructed gap (Fig. 4).

The pigeons also displayed a statistically significant tendency to fly through the right-hand gap (GLME: *z*=2.13, *P*=0.03), which they took on 65% of the 105 flights (overall odds of selecting the right-hand gap: 1.84; marginal odds of selecting the right-hand gap: 1.67 when unobstructed, 2.86 when obstructed). Because the home loft was located to the left of the exit, it follows that the birds were not choosing the gap that was most closely aligned with their intended flight direction. Instead, they appear to have been flying with a specific handedness. Importantly, the direction of this handedness was not universal to all birds: some individuals displayed a strong preference for flying towards the right-hand side independent of obstacle placement (e.g. pigeons B01, B65, S57, X41), as indicated by marginal odds ratios significantly larger than 1, whereas a smaller number of individuals (e.g. pigeons D10, X53, C68) displayed a strong preference for flying to the left independent of obstacle placement, with marginal odds ratios significantly below 1 (Fig. 3). There is also a possibility that the right preference observed was in response to the left side of the room behind the release point being more cluttered. For example, the computer controlling the motion capture system was located in the far-left corner of the room. Because the birds were released 2 m ahead of the wall, the placement of this equipment would not have interfered with the appearance of their frontal visual field, but their peripheral vision is such that the computer station would still have been in sight.

### Simulations of steering behaviour

The birds tended to fly upwards from the release point, with a mean altitude gain of 0.73±0.48 m. The mean transverse clearance of the pigeons from the curtain was 0.47±0.30 m, or approximately two-thirds of an average wingspan, with 0.80±0.48 m mean transverse clearance from the wall. Although the pigeons therefore tended to fly closer to the curtain than to the wall, the fit of the guidance simulations with parameters fitted independently to each flight was closer when the target was defined as the midpoint of the gap than when the target was defined as the point 0.35 m from the edge of the curtain (Wilcoxon signed rank test: *z*=–2.17, *P*=0.030; test of mean RMS error over all six guidance models fitted independently to each flight). The quantitative results reported below (see Dataset 1 for summary) are therefore for simulations targeting the midpoint of the gap, which is the same definition used in previous work on pigeon gap-steering behaviour (Lin et al., 2014). The quantitative results of the simulations targeting the point 0.35 m from the edge of the curtain are provided as Dataset 2, and are qualitatively similar in the patterns that they display.

### Guidance parameters fitted independently to each flight

**Fig. 5.**
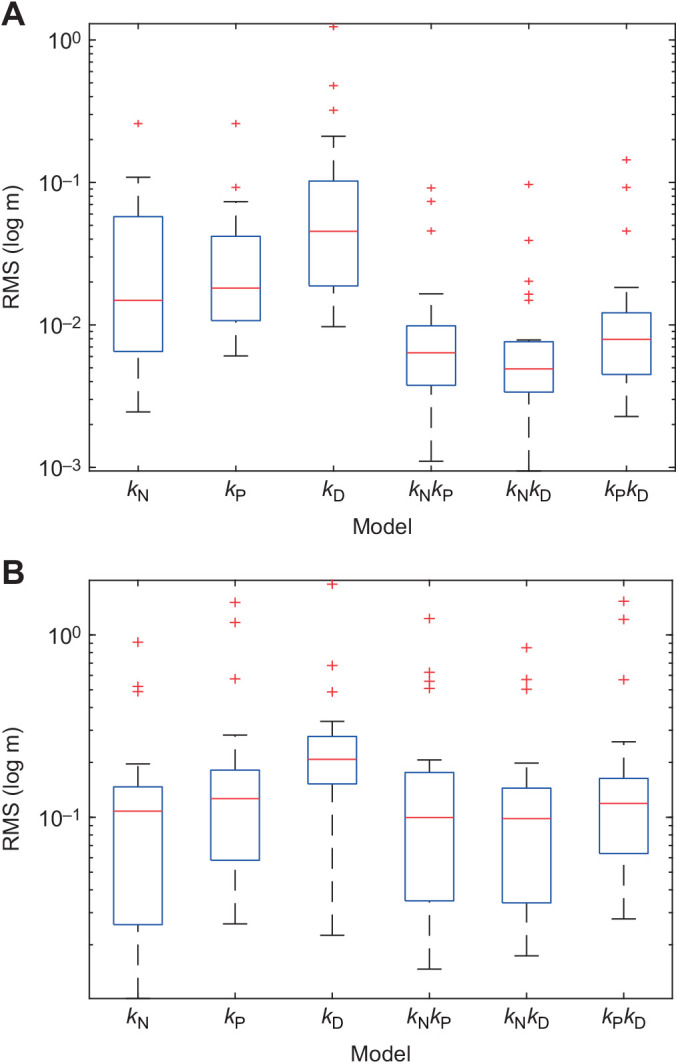
**Box plots showing root mean square (RMS) error (**ε**) of simulations under each fitted guidance model.** Results are shown for the subset of *n*=23 flights from *N*=10 individuals with ≥1 s of continuous recording in which the pigeons were tracked to within ≤1 m of the unobstructed gap through which they then flew. Each simulation is fitted to data from only the last 1.0 s of the flight. (A) Models with parameters fitted independently to each of the flights. (B) Models with parameters fitted globally to these same flights. The blue box encloses the middle half of the data, with Q1 at the bottom and Q3 at the top; the red line denotes the median; red plus signs denote outliers.

**Fig. 6.**
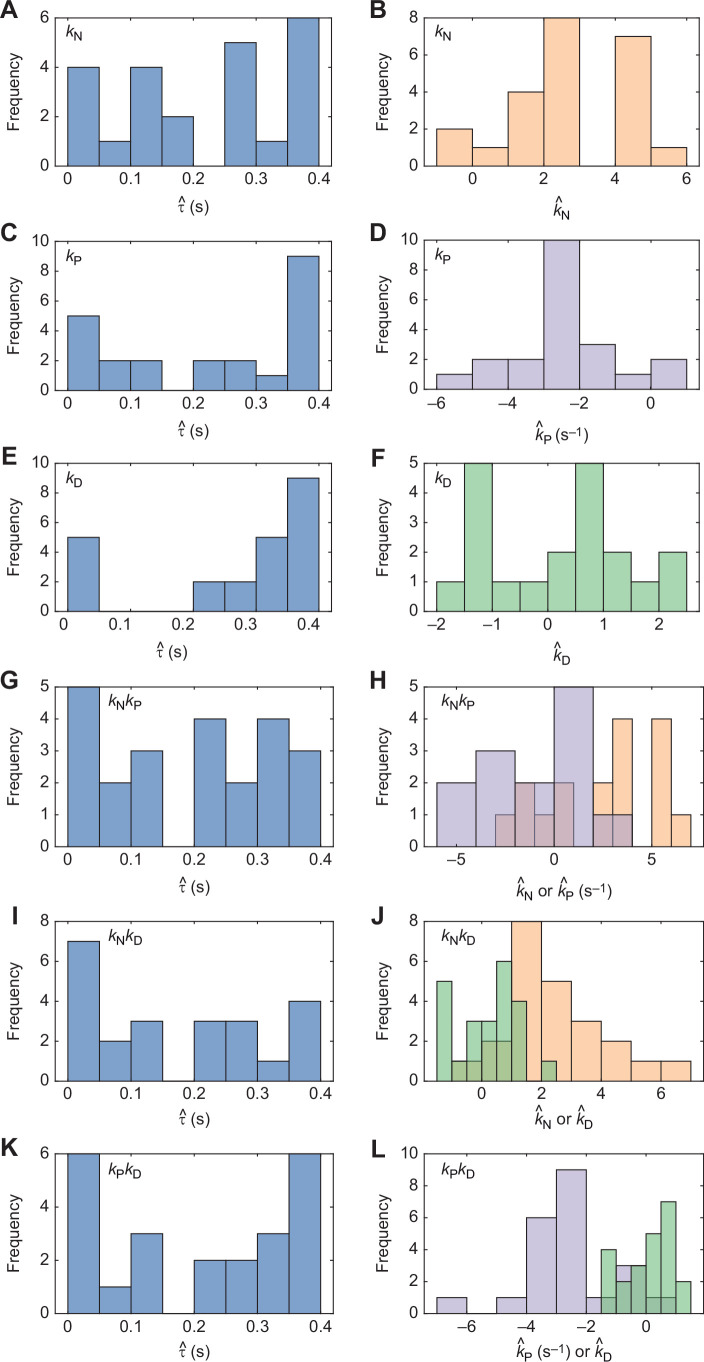
**Distributions of parameter estimates for guidance models fitted independently to each flight.** Results are shown for the subset of *n*=23 flights from *N*=10 individuals with ≥1 s of continuous recording in which the pigeons were tracked to within ≤1 m of the unobstructed gap through which they then flew. Each simulation is fitted to data from only the last 1.0 s of the flight. Fitted values of the guidance parameters are not shown for a few cases where the fitting did not converge on a value within the axis limits; see Dataset 1 for all fitted values including these outliers. Left column: histograms of parameter estimates for time delay τ (blue). Right column: histograms of parameter estimates for guidance constants *k*_N_ (orange), *k*_P_ (lilac) and *k*_D_ (green), for all *n*=23 flights. (A,B) *k*_N_ guidance; (C,D) *k*_P_ guidance; (E,F) *k*_D_ guidance; (G,H) *k*_N_*k*_P_ guidance; (I,J) *k*_N_*k*_D_ guidance; (K,L) *k*_P_*k*_D_ guidance.

All six steering controllers that we tested (Table 1) were capable of simulating the flight trajectories closely if their parameters were fitted independently to the last 1.0 s of each flight (Fig. 5A). The associated estimates of the delay spanned a broad range of values (Fig. 6), with a model average of 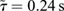 (Q1, Q3: 0.07, 0.36 s) over all six guidance laws. Among the three single-input guidance laws that we tested, *k*_N_ guidance modelled the data most closely (Fig. 5A), with a median RMS error of 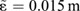 (Q1, Q3: 0.007, 0.056 m). The other single-input controllers that we tested had a higher median RMS error, with 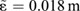 for *k*_P_ guidance (Q1, Q3: 0.011, 0.042 m) and 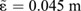 for *k*_D_ guidance (Q1, Q3: 0.020, 0.101 m), but in neither case was this difference statistically significant across flights (Wilcoxon signed rank test: *z*=0.37, *P*=0.72; *z*=1.86, *P*=0.06). As expected, our estimates of the guidance constants were consistently positive under *k*_N_ guidance (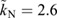; Q1, Q3: 1.9, 4.2; sign test: *P*<0.001; Fig. 6B), and consistently negative under *k*_P_ guidance (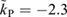 s^−1^; Q1, Q3: −3.7, −1.9 s^−1^; sign test: *P*<0.001; Fig. 6D). In contrast, they were inconsistently signed under *k*_D_ guidance (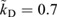; Q1, Q3: −1.0, 1.1; sign test: *P*=0.40; Fig. 6F), which reflects the over-fitting expected for a controller with no inherent tendency to steer towards a target. In summary, either proportional navigation (i.e. *k*_N_ guidance) or proportional pursuit (i.e. *k*_P_ guidance) is capable of providing a reasonable model of the data, with proportional navigation providing a marginally better fit.

Introducing a second input variable reduced the error between the measured and simulated trajectories by at least a factor of 2 (Fig. 5A). Of the various two-input controllers that we tested, *k*_N_*k*_D_ guidance provided the closest fit (Fig. 5A), with a median error of 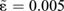 m (Q1, Q3: 0.003, 0.007 m) that was significantly lower than for the best-fitting of the single-input guidance laws (Wilcoxon signed rank test: *z*=–4.20, *P*<0.001). The other two-input controllers that we tested had a higher median RMS error, with 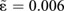 m for *k*_N_*k*_P_ (Q1, Q3: 0.004, 0.010 m) and 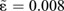 m for *k*_P_*k*_D_ (Q1, Q3: 0.005, 0.011 m). However, in neither case was this difference statistically significant across flights (Wilcoxon signed rank tests: *z*=1.00, *P*=0.32; *z*=1.43, *P*=0.15), and in both cases the fit was significantly closer than for the best-fitting of the single-input guidance laws (Wilcoxon signed rank test: *z*=–3.56, *P*<0.001; *z*=–2.43, *P*=0.015). Notwithstanding their improved fit over single-input *k*_N_ or *k*_P_ guidance, there is clear evidence that all of the two-input guidance models were over-parameterized. Specifically, the estimates of both guidance constants under *k*_N_*k*_P_ guidance were inconsistently signed (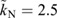; Q1, Q3: −1.6, 5.2; sign test: *P*=0.21; 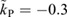 s^−1^; Q1, Q3: −5.4, 1.7 s^−1^; sign test: *P*=1.0; Fig. 6H). Therefore, there is no evidence that proportional navigation and proportional pursuit are combined under *k*_N_*k*_P_ guidance. In contrast, although the estimates of *k*_N_ and *k*_P_ remained consistently signed when fitted in combination with *k*_D_, the associated estimates of *k*_D_ were inconsistently signed (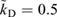 under *k*_N_*k*_D_; Q1, Q3: −0.6, 1.0; sign test: *P*=0.40; Fig. 6J; 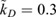 under *k*_P_*k*_D_; Q1, Q3: −0.5, 0.7; sign test: *P*=0.40; Fig. 6L). Therefore, there is no evidence that derivative control is combined with proportional navigation or proportional pursuit under *k*_N_*k*_D_ or *k*_P_*k*_D_ guidance.

In summary, when the guidance parameters were fitted independently to the last 1.0 s of each flight, proportional navigation (i.e. *k*_N_ guidance) gave the best fit of all the single-input steering controllers, but fitted the data only marginally better than proportional pursuit (i.e. *k*_P_ guidance). Whilst it was always possible to achieve a closer fit by adding a second guidance term, the parameter estimates for the second guidance term were always inconsistently signed. The guidance simulations fitted independently to the last 1.0 s of each flight therefore provide no evidence for the involvement of a second guidance term, but do not allow us to distinguish unambiguously between single-input proportional navigation or proportional pursuit.

### Guidance parameters fitted globally for all flights

**Fig. 7.**
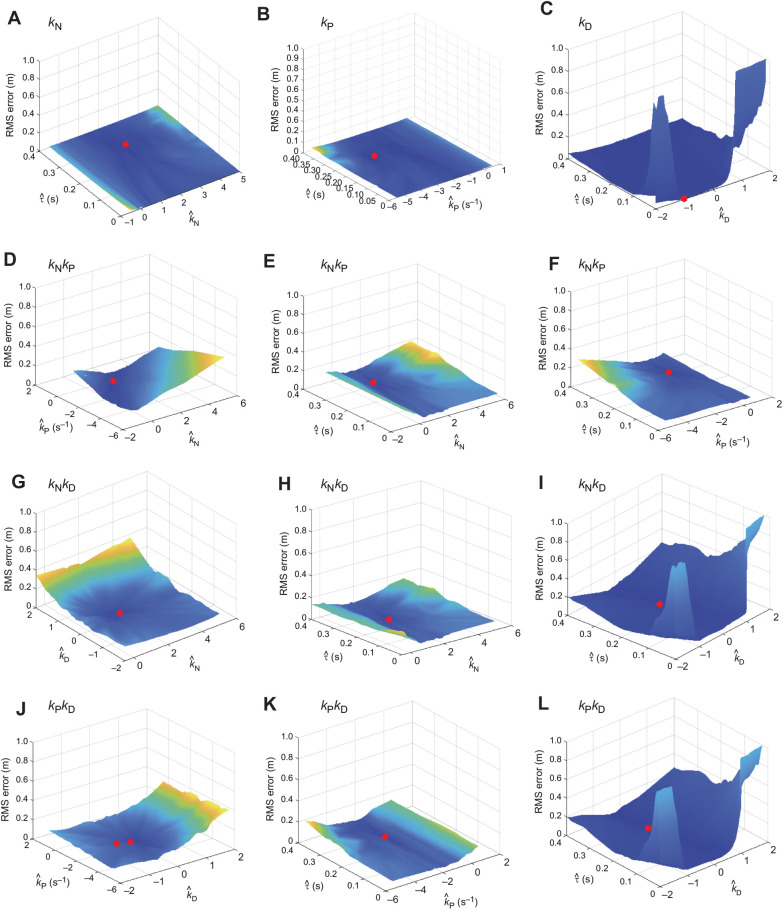
**Optimization surfaces for steering controllers fitted globally for all flights.** Results are shown for the subset of *n*=23 flights from *N*=10 individuals with ≥1 s of continuous recording in which the pigeons were tracked to within ≤1 m of the unobstructed gap through which they then flew. Each simulation is fitted to data from only the last 1.0 s of the flight. The height of each surface displays the median RMS error of the given model, as a function of the fitted guidance parameters. (A–C) Single-input guidance laws, plotting median RMS error against the relevant guidance constant (*k*_N_, *k*_P_ or *k*_D_) and time delay τ. (D–L) Two-input guidance laws, plotting median RMS error against the relevant pair of guidance constants (*k*_N_, *k*_P_ and/or *k*_D_), or their respective pairings with the time delay τ. Surface colour scaled according to surface height; red circle denotes location of global minimum.

Fitting the guidance parameters independently to each flight is appropriate if the true underlying guidance gains vary within or between individuals (Brighton et al., 2017), but risks over-parameterisation otherwise. To mitigate this risk, we searched for the unique combinations of parameter settings (Table 2) that minimised the median RMS error for each candidate guidance law over the last 1.0 s of all *n*=23 flights (Fig. 7). We did this using an exhaustive search procedure in which we computed the RMS error on every flight, for all combinations of *k*_N_∈[−0.5, 5.5], *k*_P_∈[−5.5, 0.5] and *k*_D_∈[−2, 2] at 0.1 spacing, and τ∈[0, 0.4] at 0.005 spacing (SI units). We chose these intervals in light of the ranges of the parameter estimates that we had identified by fitting all flights independently, and used the resulting simulations to identify the global optimum minimising the median RMS error over all flights. Having identified this global optimum at coarse parameter spacing, we then refined the search spacing by a factor of 5 in the vicinity of the coarse global optimum. Fitting the parameters of these steering controllers globally for all flights reduced the total number of fitted parameters by a factor of 23, but also increased the median RMS error by an order of magnitude (Fig. 5B).

**Table 2.**
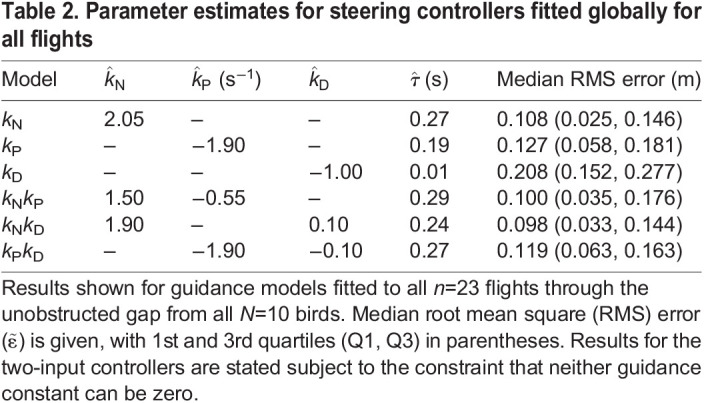
Parameter estimates for steering controllers fitted globally for all flights

Among the three single-input steering controllers that we tested, *k*_N_ guidance once again provided the best fit to the data, with a median RMS error of 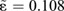 m (Q1, Q3: 0.026, 0.144 m; Fig. 5B). The parameter estimate for the navigation constant in this globally fitted model (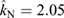) was somewhat lower than the median of the parameter estimates for the navigation constants fitted independently to each flight (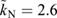), but well within the typical range of fitted values (Q1, Q3: 1.9, 4.2). The associated parameter estimate for the time delay 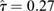 s was also in the middle of the range of values fitted independently to each flight (see above). The other single-input controllers that we tested performed significantly less well than *k*_N_ guidance when fitted globally to all fits (Fig. 5B), with a median RMS error of 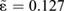 m for *k*_P_ guidance (Q1, Q3: 0.058, 0.178 m; Wilcoxon signed rank test: *z*=2.13, *P*=0.033) and 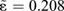 m for *k*_D_ guidance (Q1, Q3: 0.156, 0.275 m; Wilcoxon signed rank test: *z*=2.83, *P*=0.005).

Adding a second input variable produced only a marginal improvement in the goodness-of-fit of the globally fitted models (Table 2; Fig. 5B). Indeed, although *k*_N_*k*_D_ guidance once again emerged as the best of the two-input models, with a median RMS error of 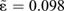 m (Q1, Q3: 0.035, 0.140 m), it provided no statistically significant improvement in fit over single-input *k*_N_ guidance in the globally fitted analysis (Wilcoxon signed rank test: *z*=–0.90, *P*0.37). The results of the globally fitted guidance models therefore confirm without ambiguity that single-input proportional navigation is the best supported of the six alternative steering controllers when fitting these to the last 1.0 s of each flight.

### Extension of model fitting to entire measured trajectory

**Fig. 8.**
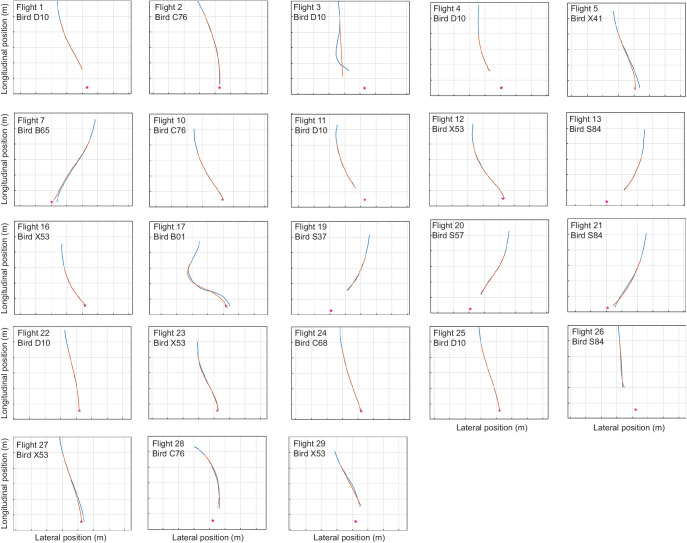
**Trajectory simulations of gap-oriented steering in pigeons under proportional navigation guidance.** Results are shown for the subset of *n*=23 flights from *N*=10 individuals with ≥1 s of continuous recording in which the pigeons were tracked to within ≤1 m of the unobstructed gap through which they then flew. Measured two-dimensional trajectory data are shown in blue; the corresponding simulations under delayed *k*_N_ guidance are shown in orange. Each simulation begins from the start of the recording, using the values of the navigation constant *k*_N_ and time delay τ from the simulations fitted to the last 1.0 s of each flight independently, treating the midpoint of the gap (red star) as the target. Note that the simulations plotted here are initiated from time *t*=τ after the start of the recorded trajectory, so begin from an earlier point and cover more of the data than that to which they were fitted, which guards against over-fitting of the simulations to their initial conditions. Flight numbers and bird identity codes correspond to those in Datasets 1 and 2. Grid spacing: 1.0 m.

Having identified delayed proportional navigation targeting the midpoint of the gap as the best supported of the six candidate guidance laws over the last 1.0 s of each flight, we finally used the same model to simulate the entire measured length of the same *n*=23 trajectories, from time *t*=τ to the end of the flight (Fig. 8). Because the values of the navigation constant *k*_N_ and time delay τ were inherited from the simulations that we had already fitted over the last 1.0 s of each flight (Fig. 6A,B), they are no longer expected to be strictly optimal, and would only be expected to result in a close fit to the data if the birds were engaged in consistent gap-oriented steering behaviour for the entire duration of the measured trajectory. This appears to be the case in general, because with only a few exceptions, the simulations still capture the curvature of the measured flight trajectories closely (Fig. 8), with a median RMS error of 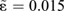 m (Q1, Q3: 0.007, 0.072 m).

## DISCUSSION

Our analysis shows that the horizontal flight trajectories of pigeons steering towards vertical gaps are best modelled by the same proportional navigation guidance law that best models falcons attacking stationary or manoeuvring targets (Brighton et al., 2017; 2021a,b). There are several lines of evidence that support this conclusion. Firstly, proportional navigation (i.e. *k*_N_ guidance) fitted the trajectory data more closely than any other single-input steering controller that we tested in simulations fitting the guidance parameters independently to each flight, albeit not significantly so at α=0.05. Secondly, proportional navigation fitted the data significantly more closely than any other single-input guidance law that we tested in simulations fitting the guidance parameters globally for all flights. Thirdly, adding a second input to this proportional navigation controller (i.e. adding either a *k*_P_ or *k*_D_ guidance term) resulted in a marginal and non-significant improvement in fit when the parameters were fitted globally to all flights, and was associated with inconsistently signed parameter estimates. Our results further show that the pigeons' steering behaviour is significantly better modelled by treating the target of its guidance as the midpoint of the gap, rather than the point falling approximately half a wingspan from the nearside edge. This result holds on average across all of the guidance models that we fitted independently to each flight, and also holds for the proportional navigation controller in particular – albeit that the qualitatively better fit of the model treating the midpoint of the gap as the target of the bird's guidance is not quite significant in this specific case (Wilcoxon signed rank test: *z*=–1.89, *P*=0.059).

Our results therefore support Lin et al.’s (2014) conclusion that feeding back the rate of change of the deviation angle (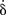) through derivative control (*k*_D_) is unimportant to modelling gap steering behaviour in pigeons. However, we also show that this behaviour is better modelled by a proportional navigation (*k*_N_) controller feeding back the line-of-sight rate (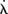) of the midpoint of the gap than by Lin et al.'s (2014) original model of a proportional pursuit (*k*_P_) controller feeding back the deviation angle of the midpoint of the gap (δ). This conclusion has implications for our assumptions regarding the underlying sensory mechanism. Specifically, whereas measurement of the deviation angle (δ) is expected to require knowledge of the retinal coordinates of the target and angular position of the head with respect to the body or its velocity, measurement of the line-of-sight rate (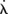) is expected to require knowledge of the retinal drift rate of the target and angular rate of the head in an inertial frame of reference. The sensory requirements of these two guidance laws are therefore quite different, although in principle, either could be used to model the data satisfactorily. More importantly, our results confirm the possibility of uniting the study of target-oriented steering behaviours of all kinds under one common algorithmic framework. Specifically, we have now shown that the same proportional navigation guidance law that best models attack behaviours in falcons (Brighton et al., 2017, 2021a,b) and that successfully models the pursuit behaviour of some hawks (Brighton and Taylor, 2019) also provides the best model of gap-oriented steering behaviour in pigeons. This is consistent with our emerging hypothesis that target-oriented guidance behaviours of birds of all kinds share a common evolutionary origin deep in their phylogeny (Brighton and Taylor, 2019).

In fact, our numerical estimates of the navigation constant *k*_N_ in the proportional navigation simulations fitted independently to the obstacle avoidance flights of our pigeons (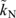
=2.6; Q1, Q3: 1.9, 4.2) were quantitatively similar to those found previously in peregrine falcons attacking stationary targets (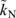
=2.6; Q1, Q3: 1.7, 3.3). This has several further implications, which we elaborate here utilising some classical results describing the behaviour of proportional navigation at different values of the navigation constant *k*_N_ (Shneydor, 1998). The statistically significant result that 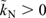 in the guidance models fitted independently to each flight (one-tailed sign test: *P*<0.001) confirms that the pigeons engaged in target-oriented steering behaviour immediately before passing through the gap, because proportional navigation produces turning towards a stationary target at *k*_N_>0. Likewise, the statistically significant result that 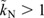 in these simulations (one-tailed sign test: *P*<0.001) confirms that the pigeons were not using any form of pure or deviated pursuit, which is the flight behaviour that proportional navigation describes at *k*_N_=1. More generally, proportional navigation produces a turn of ever-decreasing radius against a stationary target at *k*_N_<2, with a turn of constant radius produced at *k*_N_=2. Hence, the statistically significant result that 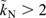 in the guidance models fitted independently to each flight (one-tailed sign test: *P*=0.047) confirms that the pigeons usually made a turn of increasing radius as they approached the gap. This is an appropriate behaviour for flight aimed at a narrow gap, because it causes flight to straighten out as the bird passes through the gap, avoiding the need for manoeuvring when flying through the confines of the gap itself, and ensuring visibility of any obstacles beyond it. Proportional navigation at *k*_N_>2 may therefore be a better guidance law for negotiating a narrow gap than proportional pursuit, which produces a tightening turn. Indeed, the median value of 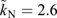 that we found is not significantly lower (sign test: *P*=0.11) than the theoretical optimum of *k*_N_=3 that commands the most efficient turn towards a stationary target, in the sense of minimising the control effort measured by the time integral of the squared acceleration command.

It is interesting to note that the goblet-shaped spatial distribution of our pigeons' flight trajectories (Fig. 4) closely resembles the results of experiments from flies and locusts presented with a choice between two spatially distinct options (Sridhar et al., 2021). In both these cases, the animal appears to adopt the average path between the options until some critical phase transition occurs, at which point the system decides spontaneously between them. Our pigeons likewise displayed a centered response initially, flying forwards from the point of release, before turning in the direction of one of the two gaps and then steering through it (Fig. 4). Theoretical modelling of this two-option spatial decision-making process (Sridhar et al., 2021) associates the moment at which turning begins with the timing of the decision to turn towards one or the other option, but the results of our guidance modelling suggest a more nuanced interpretation in the case of our pigeons. Specifically, because the line-of-sight rate of a stationary target will only be non-zero if the subject is moving, it follows that the subject must already be moving in order to generate a steering command under proportional navigation. In the presence of sensorimotor delay, this means that the animal may not begin turning at all until after it has begun moving. Hence, whereas proportional navigation can only produce monotonic turning towards a stationary target in the absence of delay, it can produce a sinuous turn in the presence of a delay. This is already sufficient to explain the sinuous nature of some of the measured trajectories plotted in Fig. 8. Nevertheless, as these trajectories do not begin from the moment of take-off, it remains possible that the pigeons did not make their targeting decision until after they had already begun flying forwards from the release point. This would be consistent with Lin et al.’s (2014) argument that pigeons rely on short-range guidance, based on the observation that the pigeons in their study showed no evidence of steering toward gaps until 1.5 m (*ca.* 0.33 s) before encountering vertical clutter. Any such delay in their decision making may also explain why the pigeons did not show any clear preference for flying through the unobstructed gap (Fig. 3). This is because the obstacle was placed between the release point and the gap (Fig. 2), so may not have obstructed either gap from the perspective of the subject at the point where its targeting decision was finally taken (Fig. 4).

The preceding account points to the importance of determining both the outcome and the timing of targeting decisions when modelling movement through complex environments. In cluttered environments requiring multiple targeting decisions, the timing of every decision will be critical to determining the overall shape of the trajectory (Lin et al., 2014). The general question of how a chain of spatial decisions structures trajectories through complex environments clearly merits further investigation, because the portion of a trajectory over which steering can occur under closed-loop guidance is obviously limited to the interval between the point at which a targeting decision is made and the point at which the animal reaches its target. Fast decision making is therefore critical when flying through clutter, and open-loop turning commands may be important to ensuring that flight is directed appropriately during the period of any delay between target acquisition and the onset of closed-loop guidance. One method of increasing the speed of decision making when choosing between similar alternatives is to introduce a bias in the decision-making process. There is clear evidence of biased choice in our data, because at the population level our birds displayed a clear preference for selecting the right-hand gap, whereas at the individual level most birds displayed their own idiosyncratic preference for choosing the right or left gap. This is consistent with individual biases observed in budgerigars choosing between two apertures of differing sizes (Bhagavatula et al., 2014). We speculate that such biases may aid decision making when flying through clutter, which is a situation in which making and committing to a decision quickly may be at least as important as the decision that is made (Lin et al., 2014). This hypothesised enhancement of speed and safety when flying through clutter has been shown to be advantageous for flocks of birds as well as individuals (Bhagavatula et al., 2014), but how might such biased choices arise? There is widespread evidence of handedness in animals (Warren, 1980), which has already been shown to accelerate homing over longer distances in swimming and flying animals (Bandyopadhyay et al., 2013). Handedness can be of mechanical, sensory or neural origin, but based on the information available here, we cannot distinguish whether the biased choice that we observed reflects the intrinsic handedness of different individuals or an idiosyncratic response to extrinsic cues. For instance, as the preference to fly down the right-hand side of the hall continued after the pigeons had passed through the gap, it is plausible that individual birds simply used their visual memory to recapitulate the fine-scale route that they had followed on their initial release, as has already been shown in large-scale homing behaviours (Biro et al., 2006).

Finally, the guidance framework that we have applied has implications for understanding the sensory information that animals use to structure their goal-directed behaviours. The steering behaviour that we observed in our pigeons was best explained by assuming that they targeted the midpoint of the gap (which would maximise the clearance on both sides), rather than the point half-a-wingspan in from the near edge (which would have minimised the nearside clearance with the wings spread). It is therefore reasonable to assume that this behaviour would not have required metric estimation of the physical width of the gap. In contrast, as the midpoint of the gap represents a virtual target, how its line-of-sight rate (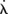) would have been estimated by the birds remains an open question. One possibility is that the birds could have centred the gap in their visual field, and estimated its line-of-sight rate from the angular rate of their head by integrating the angular accelerations sensed by their vestibular system. Another possibility is that they could have made use of the rotational optic flow cues produced by the head's self-motion relative to a fixed visual background. A gap-centering response of this kind would differ from the mechanism observed in birds avoiding single obstacles, which appear to fixate their gaze on the edge of the obstacle that they are aiming to avoid (Kress et al., 2015; Miñano et al., 2023). This again raises the question of whether animals perceive clutter as a set of gaps to be aimed at or a set of obstacles to stay clear of. Establishing a guidance model for gap-oriented steering behaviour in pigeons, as we have done here, reduces the problem to one of determining how and when they select which clearances to fly through.

### Limitations

Although the number of individuals that we tested was twice that of other similar studies (Bhagavatula et al., 2014; Lin et al., 2014), our sample size is small in absolute terms, and involves repeated measures from the same individuals that could not always be controlled for statistically. It follows that our results cannot necessarily be assumed to generalise more broadly. The sections of flight that were fitted under the guidance models are also quite short, which is inevitable when modelling flight through clutter, but explains why the observed flight trajectories can often be modelled successfully by more than one guidance model. Hence, although proportional navigation was the best supported of the various guidance models that we tested in pigeons negotiating a single gap from a choice of two, it is plausible that a different result might emerge given a larger sample size, a longer flight duration or a greater number of gaps. Future studies could usefully increase the total number of individuals tested, the distance between the release point and the gap, and the number of gaps, explicitly testing the role of handedness in the context of an experimental setup more like the forest of vertical obstacles employed by Lin et al. (2014). Finally, it is important to note that the experimenter who released the birds by hand could not be made blind to the experimental condition (Fig. 2). However, as there was no evidence of any effect of obstacle placement on gap choice, this is unlikely to have affected the experimental results. Other possible biases such as the handedness of the experimenter would have affected different birds similarly, so are unlikely to explain their idiosyncratic preference for choosing the right or left gap.

## Supplementary Material

10.1242/jexbio.244215_sup1 Supplementary information
