# Supplementary material for: Gap selection and steering during obstacle avoidance in pigeons
Source: J Exp Biol. 2023 Jan 23;226(2):jeb244215. doi: 10.1242/jeb.244215 (PMC10086542; doi:10.1242/jeb.244215)
Supplement: Supplementary information [file jexbio-226-244215-s1.pdf]

Dataset 1 contains the results of simulations treating the midpoint of the gap as the target of the bird's guidance.

Click here to download Dataset 1

Dataset 2 contains results of simulations treating the point 0.35 m from the edge of the curtain as the target of the bird's guidance.

Click here to download Dataset 2

Journal of Experimental Biology • Supplementary information
